# Potential Active Marine Peptides as Anti-Aging Drugs or Drug Candidates

**DOI:** 10.3390/md21030144

**Published:** 2023-02-23

**Authors:** Hui Yang, Qiting Zhang, Bin Zhang, Yufen Zhao, Ning Wang

**Affiliations:** 1 Institute of Drug Discovery Technology, Ningbo University, Ningbo 315211, China; 2 College of Chemistry and Chemical Engineering, Xiamen University, Xiamen 361005, China; 3 Li Dak Sum Yip Yio Chin Kenneth Li Marine Biopharmaceutical Research Center, Department of Marine Pharmacy, College of Food and Pharmaceutical Sciences, Ningbo University, Ningbo 315800, China

**Keywords:** anti-aging drugs, multi-omics aging characteristics, aging mechanisms, marine organisms, active peptides

## Abstract

Aging is an irreversible physiological process in the human body, and the aging characteristics of the body that accompany this process also lead to many other chronic diseases, such as neurodegenerative diseases represented by Alzheimer’s disease and Parkinson’s disease, cardiovascular diseases, hypertension, obesity, cancer, and so on. The marine environment is highly biodiverse, the natural active products of these organisms constitute a vast treasure trove of marine drugs or drug candidates that play an essential role in disease prevention and treatment, and the active peptide products among them have received special attention because of their unique chemical properties. Therefore, the development of marine peptide compounds as anti-aging drugs is emerging as an important research area. This review highlights the currently available data on marine bioactive peptides with anti-aging potential from 2000 to 2022 by analyzing the prevalent aging mechanisms, critical aging metabolic pathways and well-established multi-omics aging characteristics, as well as grouping different bioactive and biological species lines of peptides from marine organisms and discussing their research modalities and functional characteristics. Active marine peptides is a promising topic to explore and to develop their potential as anti-aging drugs or drug candidates. We expect this review to be instructive for future marine drug development and to reveal new directions for future biopharmaceuticals.

## 1. Introduction

Aging is becoming the greatest risk factor for most chronic diseases, leading to increased morbidity and mortality in elderly patients. Understanding the mechanism of aging and paying attention to the related hallmark features of aging are beneficial to delaying aging and prolonging healthy life at the overall level of the body, which is a necessary condition for delaying aging and developing new anti-aging drugs in the future [1]. In modern biology, the hallmarks of aging satisfy the following three prerequisite characteristics: (1) age-related manifestations, (2) the experimental acceleration of aging, and (3) therapeutic interventions that slow, prevent, or reverse aging [2]. A variety of aging theories are based on various features of aging, such as the free radical theory, the immune theory, the telomere theory, the hormone theory, and the cellular aging theory [3]. Meanwhile, aging is often regulated by specific signaling pathways (e.g., AMPK, SIRT1, and mTOR pathways), which can act both individually and in combination to influence the aging process [4].

Aging is not a disease [5], and anti-aging medicine aims to slow, stop, or reverse the aging process and its associated effects, such as disability and frailty. Currently, anti-aging drugs, such as resveratrol, rapamycin, metformin and aspirin, mainly target aging characteristics that affect autophagy and inflammation. They have shown effectiveness in extending healthy lifespan and aging retardation in animal models of aging [6]. In addition, hormone mimetics, such as estrogen, progesterone, testosterone, and DHEA, are widely used in elderly populations to improve the various symptoms of aging associated with frailty, body composition, cardiometabolic diseases, neurodegenerative diseases, and quality of life. In recent years, the composition of gut microbiota associated with aging [7] has also demonstrated great anti-aging potential. However, given that most of the currently used anti-aging drugs are traditional “small molecule” drugs with low target selectivity or “protein agents” with low bioavailability, it is becoming increasingly popular to search for peptide drugs that combine the advantages of small molecules and proteins [8]. As a natural ecosystem rich in unknown organisms, life in the marine environment can produce numerous compounds of various molecular weights. Marine peptides have not only shown excellent biological activities in the field of drug development, such as anticancer, anti-bacterial, anti-viral, anti-oxidant, immunomodulatory, and neuroprotective [9,10], but also have the potential benefits of the innate blocking of protein interactions, low immune response, high chemical and biological diversity, and low toxicity; therefore, the isolation of active peptides from marine organisms [11] for the prevention and treatment of aging and its related diseases is a very promising strategy for drug development.

To date, multiple bioactive peptides from underutilized marine resources have affected various aging markers through various mechanisms at different molecular levels, showing non-negligible anti-aging potential properties [12]. Currently known marine peptides have shown various mechanisms of action that interfere with aging [13], such as improving anti-oxidant or anti-fatigue capacity [14], enhancing mitochondrial function [15], and enhancing insulin sensitivity [16]. In addition, they are also able to improve immune levels, inhibit lipid peroxidation, inhibit cell apoptosis, inhibit telomere loss, and delay cell aging, etc. [17,18]. This demonstrates the inestimable potential of marine peptides for anti-aging drug research and development. However, although hundreds of compounds have been shown to extend the lifespan of experimental model organisms [19], current research on natural marine peptides as anti-aging drugs is still limited. Most of the current research on marine peptides focuses on skin anti-aging in the cosmetic industry [20] and almost all the anti-aging drugs that have been marketed are also natural small molecule compounds [6]. Based on the above background, this review mainly reveals the current research status of marine active peptide compounds that intervene in aging mechanisms and critical pathways by regulating key markers of aging and aging characteristics. Using marine ecology as the research environment, this review focuses on a selection of peptides obtained under different extraction or isolation methods and explores the anti-aging activity and possible therapeutic potential of these compounds exhibited in biochemical experiments and biochemical analyses by means of multi-omics functional analysis, physiological tests, theoretical calculations, and virtual predictions. This work provides useful information for the future development of these functional bioactive peptides as anti-aging drugs or drug candidates and for the extension of a healthy lifespan in humans.

## 2. The Mechanism of Aging under the Theory of Aging

### 2.1. The Free Radical/Oxidative Stress Theory

The free radical/oxidative stress theory, which first emerged in 1956, states that the aging of organisms occurs as a result of the accumulation of oxidative damage and stress from reactive oxygen species (ROS). Under normal physiological conditions, a balance is maintained between the production and scavenging of reactive oxygen species in organisms; however, when the production of ROS exceeds the scavenging capacity of the organism, the excessive accumulation of ROS can cause damage to nucleic acids, lipids, and proteins, leading to cellular damage and death [21], thus triggering aging and age-related diseases. There is now critical evidence that the in vitro supplementation of anti-oxidants protects cells from various types of damage and has shown some efficacy in mouse models of aging, AD models, or limited clinical trials of human AD [22]. Therefore, the inhibition of excessive oxidative stress by anti-oxidant compounds to achieve the goal of lowering the level of oxidative stress in organisms is considered by many authors as a promising intervention strategy to delay or prevent age-related pathological cases [23].

### 2.2. Immunological Theory

The immune theory of aging, proposed in 1989, states that the function of the immune system of the biological organism gradually declines with age, affecting the resistance of the organism to disease. The immune system plays a fundamental role in the elimination of senescent cells, and the decline in immune function is associated with an increase in the number of senescent cells, which eventually leads to various diseases [24], such as cancer, human immunodeficiency virus, multiple sclerosis, and aging [25,26,27]. Thus, in the aging system, the immune response plays a key role in maintaining human health. It has been shown that protein hydrolysates [28], which are effective sources of bioactive peptides, have applications in immunomodulation [29]. Therefore, the development of anti-aging pharmaceutical agents with immunomodulatory properties may protect cells from free radical stress and ultimately exhibit anti-aging activity.

### 2.3. The Mitochondrial Theory

It is well known that mitochondria are the center of many important cellular functions and the “powerhouse” of energy and that another important physiological process that affects cellular aging and regulates biological growth, cell division, also occurs in mitochondria, demonstrating the importance of mitochondria in the biological growth process. The accumulation of decreased mtDNA abundance during cell division and altered mitochondrial dynamics as aging progresses may lead to a deterioration of mitochondrial function, ultimately leading to increased ROS production and altered mitochondrial membrane permeability, resulting in inflammation and cell death [30]. Current and growing evidence from preclinical models and humans suggests that metformin promotes mitochondrial fission to improve mitochondrial function and may reduce the risk of diseases associated with aging (e.g., neurological degeneration and cancer) [31]. Therefore, pharmaceutical agents targeting mitochondria that improve or restore mitochondrial function may be potential targets for the development of anti-aging drugs.

### 2.4. Hormonal Theory

Hormonal theory states, firstly, that many of the diseases of aging resemble the physical consequences of hormone deficiency and may be caused by hormone deficiency. Secondly, most of the possible causes of aging, such as excessive free radical formation, glycosylation, protein cross-linking, imbalance in the apoptotic system, etc., may be attributed to hormone deficiency. Additionally, at the genetic level of aging, such as the restriction of cell proliferation, undesirable genetic polymorphisms, and premature telomere shortening, may be associated with hormone deficiency. Thus, aging and hormones are closely related: endocrine factors influence aging, while aging alters hormonal status [32]. Hormone interference has been shown to successfully increase lifespan in several mammalian species. These interventions include the inhibition of mTOR targets and making GH/IGF-1 signaling defective [33]. The evolutionarily conserved insulin and insulin-like growth factor (IGF) signaling pathways (IIS) have been shown to play an important role in controlling longevity in invertebrates, mammals (several mouse models), and human longevity studies [34]. Findings suggest that IIS inhibitors could be used as new drugs for the treatment of neurodegenerative diseases, including AD and HD [35]. Thus, inhibitors or activators that control metabolic pathways associated with aging can be used as potential anti-aging agents for the prevention and treatment of aging as well as related diseases.

### 2.5. Gut Microbes and Aging

The stability of the coexistence between host and gut microbiota has been found to be associated with aging and longevity. In recent years, the key role of the gut microbial community residing in the gastrointestinal tract in regulating health status and longevity has been demonstrated [36]. Gut microbes interact with their hosts in several ways; on the one hand, the disruption of the normal balance of microbial populations in the gut can lead to inflammation [37], which may affect the development of the intestinal immune system [38,39]. On the other hand, it may also affect gastrointestinal dynamics and the function of certain enteric neurons [40], which may then affect the brain–gut axis [9]; in addition, it may also affect host metabolism [41]. Indeed, alterations in gut microbiota composition (sometimes referred to as “dysbiosis”) have been linked to the pathogenesis of many diseases, such as geriatric syndromes and cardiovascular diseases, e.g., atherosclerosis [42]. Thus, dietary and probiotic interventions targeting microbial communities have been shown to favorably influence host health and aging by enhancing anti-oxidant activity, improving immune dynamic balance, suppressing chronic inflammation, regulating fat deposition and metabolism, and preventing insulin resistance [43]. It reminds us that peptides could perhaps serve as potential anti-aging agents to regulate the composition of the gut microbiota and thus improve health and the potential anti-aging agents that affect a healthy lifespan [44].

**Figure 1:**
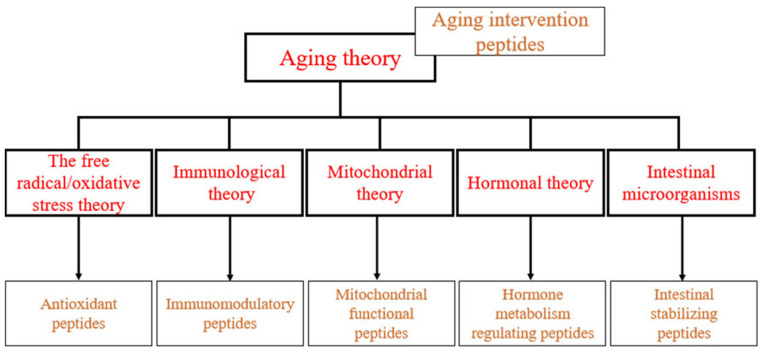
Intervention peptides within the aging theory.

Over the last few decades, researchers have demonstrated the possibility of using drugs to modulate the biological properties of aging by various biochemical means, thus “restoring” the physiological functions of complex model organisms. In this review, we summarize the discovery and recent research progress of anti-aging marine peptides in the marine field in the last decade (Figure 1) by distinguishing the differences between biological species (vertebrates, invertebrates, and marine plants) and experimental research techniques (biochemical experiments and in silico analysis), as well as the amino acid composition of certain important anti-aging marine peptides (see Supplementary Materials, Tables S1–S11), which contributes to the prospect of the use of abundant active marine peptides in the field of anti-aging medicine and providing a solution to the problem of limited resources and the utilization of active marine peptides. 

## 3. Marine Peptides That Intervene in the Aging Process

### 3.1. Anti-Oxidant Active Peptides

Organisms have developed several ways to protect themselves from oxidative attack to maintain normal redox levels in cells, including the synthesis of anti-oxidants and anti-oxidant enzymes to scavenge free radicals, inhibit lipid peroxidation, or enhance anti-oxidant enzyme activity. Therefore, peptide drugs supplemented with anti-oxidant analogs to reduce the oxidative stress of the organism and improve anti-oxidant capacity suggest a promising intervention strategy for aging, thus potentially delaying or preventing age-related pathologies [23]. To date, a comprehensive summary of a large number of studies in the literature has shown that a significant number of marine biopeptides have performed well in terms of anti-oxidant activity, and these marine-sourced anti-oxidant active peptides are potentially powerful candidates for anti-aging drugs. Table 1 summarizes the marine bioactive peptides from different biological lineages that have been identified in the current literature as having anti-oxidant activity and thus the potential to interfere with aging.

#### 3.1.1. Marine Vertebrates

First, protein hydrolysates from marine fish can form a variety of peptides with different activities [45], and the current status of research on some of these peptides that affect lifespan due to their anti-oxidant activity is as follows. One to study the effects of marine collagen peptides (MCPs) prepared from Chum Salmon (Oncorhynchus keta) skin on the lifespan and spontaneous tumor incidence in SD rats; it was found that MCPs not only inhibited the age-related decrease in anti-oxidant enzyme activity and increase in lipid peroxidation product levels in SD rats in a dose-dependent manner but also significantly extended the mean lifespan, last 30% survivor lifespan and maximum lifespan of mice, while reducing the incidence of spontaneous tumors in the rats [46]. Then, several peptides were studied after the ultrafiltration of defatted round scad (Decapterus maruadsi) protein hydrolysate, and finally, the P5 peptide (ILGATIDNSK) (1153.4 Da) with optimal anti-oxidant capacity was identified and analyzed, and it was found that the *Caenorhabditis elegans (C. elegans)* model fed with P5 peptide had a longer lifespan, higher survival rate, and higher anti-oxidant enzyme activity compared with the control group, suggesting that this class of fish protein peptides possessed good anti-aging effects [47]. In addition, we investigated deep red snapper, whose scales are rich in collagen, and obtained crimson snapper scale peptides (CSSPs) (<3000 Da) through the enzymatic hydrolysis of their scales. The results showed that CSSPs played an anti-oxidant and anti-aging role [48]. Moreover, two bioactive peptide fractions (SWP-I and SWP-II) from the swim bladder of Atlantic cod (Gadus morhua) were isolated and pretreated with SWP-I and SWP-II on prematurely senescent 2BS cells induced by hydrogen peroxide (H_2_O_2_), and it was found that SWP-I and SWP-II could effectively scavenge DPPH-, HO^-^, and O^2−^; improve Fe^2+^ chelating activity; increase cell survival; and inhibit SA-β-gal activity and apoptosis rate, indicating that they are peptides with both anti-oxidant and anti-aging properties [49]. There are also studies on grass carp (Ctenopharyngodon idella) skin, where three novel peptides (PTSPL/GPGPGL/VGGAP) with known sequences derived from the proteolytic digest of grass carp skin have potent anti-oxidant activities, and although their potential health benefits need to be explored through simulated digestion and animal studies, the powerful anti-oxidant activities shown in the current study and the findings provide evidence that these three anti-oxidant peptides are useful in delaying aging or extending the life of cells. The powerful anti-oxidant activities shown in the current paper and its findings provide the value of these three anti-oxidant peptides in the study of delaying aging or extending life health span [50].

#### 3.1.2. Marine Invertebrates

In addition to peptides of vertebrate origin, represented by fish, which exhibit anti-aging potential, there are some invertebrates that also show potential to intervene in aging due to their anti-oxidant activity. First, jellyfish collagen hydrolysate (JCH) was investigated and JCH was administered to a D-galactose-induced aging model in mice. The climbing endurance test, anti-fatigue test, and redox substance level assay revealed that JCH increased SOD activity and GSH-Px activity and had anti-oxidant effects on aging mice, which in turn significantly alleviated fatigue in mice [51]. Another peptide found to exert anti-fatigue effects due to anti-oxidant activity is an oyster peptide OH-I (<6 kDa), obtained from oyster hydrolysate (OH), which was adaptively fed to mice with OH-I peptide. After a fatigue swimming test and biochemical index measurements, it was found that the oyster peptide increased exercise endurance, free radical scavenging ability, and chemical anti-oxidant activity, showing the anti-oxidant activity and anti-fatigue effects [14].

Sea cucumbers have long been regarded as a marine food delicacy and traditional medicine, and their health benefits are due in the main to their protein content [52,53]. Sea cucumber-derived peptides are potential candidates for anti-aging [54]. Take, for instance, a study concerning the preventive and delaying effects of peptides from sea cucumber (Cucumaria rondose) hydrolysate (CFH) in aging Drosophila and D-galactose-induced aging mice with neurodegenerative diseases. The researchers found that CFH effectively prolonged Drosophila lifespan and improved learning and memory impairment in D-gal-induced senescent mice by upregulating the expression of the senescence suppressor gene Klotho [55], increasing superoxide dismutase and glutathione peroxidase activities, inhibiting lipid peroxidation and protein oxidation, and downregulating acetylcholinesterase activity. CFH is a potential biopeptide for the treatment of age-related diseases [56]. Secondly, the study of oligopeptides (SVH-PF/SVH-CAH-PF) from the protein hydrolysates of sea cucumber (Stichopus variegates) with the valuable organic matter revealed that the peptide also upregulated Klotho expression, activated SOD and GSH-Px expression, inhibited lipid peroxidation and protease oxidation, and ultimately protected aging Drosophila from in vivo oxidative stress, attenuated their oxidative damage, and prolonged their lifespan [54]. In addition, peptide preparations from sea cucumber (Apostichopus japonicus) (AjPH) were able to enhance nematode survival under oxidative stress conditions and reduce reactive oxygen species (ROS) levels; upregulate superoxide dismutase and catalase activities, as well as reduce malondialdehyde levels to enhance the nematode anti-oxidant defense system; and further studies have also shown that AjPH can reduce aging pigments and extend lifespan, indicating that AjPH has the ability to retard physiological aging. In addition, the anti-oxidant potential of two groups of short peptides, GF2 and GF3, was assessed by bioinformatics analysis [53].

### 3.2. Hormonal Metabolic Regulation of Peptides

Aging is a physiological process caused by metabolic disorders [57], and therefore the level of hormone levels in the metabolic process affects aging-related pathways. One of the most studied pathways is the insulin/IGF-1 signaling (IIS) pathway which is associated with aging [58,59]. The inhibition of the IIS signaling pathway is already known to extend lifespan and delay aging-related diseases in a variety of animal models, from nematodes to mice [60]. DAF-2, AGE-1, and DAF-16 are key molecules in the IIS pathway [61], where DAF-2 encodes a homolog of the mammalian insulin/IGF-1 receptor (INSR), AGE-1 encodes a mammalian phosphocreatine 3 kinase (PI3K) catalytic p110 subunit homolog, and DAF-16 is widely expressed to encode a homolog of the human fork head box O (FOXO) transcription factor. It has also been shown that the autophagy gene cascade signaling, which acts downstream of the insulin/IGF-1 signaling pathway (IIS) [62], is able to modulate the important process of autophagy that affects aging to regulate the anti-aging activity of the organism. Table 2 summarizes the potential anti-aging marine peptides from the different strains of marine organisms that influence aging-related metabolic levels by virtue of regulating certain key regulators in the IIS signaling pathway and related pathways.

#### 3.2.1. Marine Invertebrates

To date, bio-functional peptides involving anti-bacterial, anti-hypertensive, and anti-coagulant activities have been obtained from mussel protein hydrolysates [63]. First, oligopeptides obtained from mussel (Mytilus Edulis) protein hydrolysates were treated with hydrogen peroxide-induced senescent cells, and it was found that mussels significantly attenuated cellular senescence-related properties, such as loss of cell viability, cell cycle arrest, elevated β-galactosidase (SA-b-GAL) activity, and senescence-associated heterochromatin foci (SAHF) formation. At the same time, mussel oligopeptide also promoted the increase in glutathione (GSH) level and the restoration of mitochondrial transmembrane potential (DCM) and increased the transcriptional activity of peroxiredoxin 1 (PRX1), nicotinamide phosphoribosyl transferase (NAMPT), and sirtuin 1 (SIRT1). Therefore, this mussel oligopeptide may inhibit cellular senescence through redox cycle regulation and the enhancement of the SIRT1 pathway [64]. Subsequently, bioactive mussel polypeptide (MP) was again obtained from mussel (Mytilus Edulis) protein hydrolysate and pretreated with the nematode senescence model, which revealed improved anti-oxidant properties and the extended lifespan of nematodes, as evidenced by endogenous ROS levels; the accumulation of nematode senescence pigments; and reduced apoptosis. This may be possible via the downregulation of DAF-2 and the up-regulation of DAF-16 in the insulin/IGF-1 signaling pathway to prolong the healthy period of nematodes [65]. Two new peptides, SnP7 (AAVPSGASTGIYEALELR, 1805.03 Da) and SnP10 (NPLLEAFGNAK, 1173.34 Da), isolated and identified from the purple sea urchin (Strongylocentrotus nudus) gonad hydrolysate, were used to treat a nematode senescence model induced by paraquat and were found to reduce ROS levels, the expression of superoxide dismutase-3 (SOD-3), and heat shock protein-16.2 (HSP-16.2) and induce DAF-16 nuclear translocation and its gene expression. Taken together, the S. nudus peptide-induced enhancement of nematode anti-oxidant capacity is dependent on DAF-16 [66]. Then, another three novel anti-oxidant peptides, MmP4 (LSDRLEETGGASS), MmP11 (KEGCREPETEKGHR), and MmP19 (IVTNWDDMEK), were isolated from a clam (Meretrix meretrix), which is often used as a medicine and consumed as seafood, and the protective effects of these three anti-oxidant peptides were tested using *Caenorhabditis elegans* aging models; it was thus demonstrated that these peptides promote DAF-16/FOXO, a key regulator of oxidative stress and lifespan in *C. elegans* nuclear translocation and induce superoxide dismutase 3 (SOD-3) expression among anti-oxidant enzymes. Thus, three anti-oxidant peptides have the ability to extend the lifespan of *C. elegans* [67]. In addition, two new anti-oxidant peptides, D2-G1S-1 (VENAACTTNEECCEKK) and G2-G1S-2 (VEGGAACTTGGEEGCCEKK), were also identified from the Arca subcrenata, both of which increased the mean lifespan and resistance to oxidative stress in *Caenorhabditis elegans* and significantly improved the age-related physiological functions of *Caenorhabditis elegans*, as evidenced by the reduced accumulation of ROS, lipids, and lipofuscin in *Caenorhabditis elegans* with increased oxidative stress. Further experiments suggest that these two trichothecene peptides may prolong nematode lifespan by regulating aging-related genes through the modulation of the insulin/IGF-1 (IIS) pathway [68].

#### 3.2.2. Marine Vertebrates

Targeting marine fish, the hypoglycemic activity of marine fish roe polypeptide (FRP) in pancreatic islet β-cells (INS-1) and the molecular mechanism by which FRP exerts this effect were identified. In the study, FRP treatment promoted insulin secretion and cell viability, reduced apoptosis and intracellular ROS levels, and increased the activity and content of anti-oxidant-related enzymes. Additionally, the analysis of cellular signaling pathways revealed that FRP attenuated oxidative stress mainly by activating the Nrf2/ERK pathway [69].

### 3.3. Mitochondrial Functional Peptides

In the last few years, mitochondrial research has been in ongoing development in the field of aging, and the communication between mitochondria and nucleus that occurs due to mitochondrial dysfunction is a well-recognized phenomenon of aging and can be observed in long-lived species with perturbed mitochondrial electron transport chains [70]. Mitochondria can be involved in numerous aging-related characteristics [71], such as decreased stem cell function, cellular senescence, “inflammation”, metabolic regulation, the overproduction of ROS, and neurocognitive impairment [72], and thus modulating mitochondrial function can be an intervention to extend lifespan [73,74]. On the one hand, AMPK activation triggers autophagic flux when the organism is energy-depleted [75], immediately followed by an increase in cellular energy and the avoidance of senescent cellular energy depletion. On the other hand, at the level of integrated growth signals and cells, mTOR phosphorylation is a major regulator of autophagy, and the overactivation of mTOR [76] is closely associated with several aging [77]-related diseases, such as cancer, diabetes, and neurodegenerative diseases. In addition, the Bcl-2 family is an important regulator of apoptosis [78], and Bcl-2 activation is regulated by the post-translational phosphorylation of AKT, mTOR, and p70S6K [79]. In addition, the key regulator of cell survival and apoptosis is the NF-κB signaling pathway [80]. Anti-aging drugs, such as resveratrol and metformin [81], are those that work by targeting mitochondrial function [82].

In summary, targeting mitochondria for anti-aging prevention and treatment is a very promising therapeutic tool. A few marine peptide compounds that are able to improve or restore mitochondrial function by modulating the expression levels of important signaling molecules in mitochondria, thereby affecting biological processes associated with aging and exhibiting anti-aging potential, have now been identified, as summarized in detail in Table 3.

#### 3.3.1. Marine Vertebrates

The study of low molecular weight peptides obtained from fish protein hydrolysates to assess the effects of fish peptides on the expression of stress response gene patterns was proposed by Datson and Hunter [83,84,85]. The results revealed that fish peptides modulate the stress-responsive gene expression in the hippocampus (HC) after acute mild stress, including genes of the mitochondrial and oxidative stress pathways, which are associated with HPA axis regulation or mitochondrial activity, a finding critical for the regulation of oxidative stress. Thus, fish hydrolytic peptides may regulate circadian rhythms and aging processes and prevent neurodegenerative diseases. In conclusion, low molecular peptides obtained from fish hydrolysis appear to be a novel source of drugs for aging regulation through multiple mechanisms [86].

Firstly, two Ache inhibitory peptides, Pro-Ala-Tyr-Cys-Ser (PAYCS) and Cys-Val-Gly-Ser-Tyr (CVGSY), obtained from anchovy (Anchovy) (Coilia mystus) protein hydrolysate (APHs), were studied and treated with glutamate-induced PC12 cells, and both peptides were found to significantly increase cell viability; reduce lactate dehydrogenase release, reactive oxygen species (ROS) production, malondialdehyde content, and the Bax/Bcl-2 ratio; increase superoxide dismutase and GSH-px activity; and inhibit Ca^2+^ influx. The above results suggest that two fish peptides (PYCS and CVGSY) may alleviate memory impairment by inhibiting apoptosis through the suppression of ROS production and Ca^2+^ influx [87]. Next, a study using round scad (Decapterus maruadsi) hydrolysate (RSH) fed to a sleep-deprived rat model found that RSH significantly upregulated the expression of anti-oxidant-related proteins, including nuclear factor E2-related factor 2 (Nrf2) and its downstream heme oxygenase-1 (HO-1), as well as the phosphorylation of AKT in rats. In addition, RSH improved the expression of brain-derived neurotrophic factor (BDNF), cAMP response element binding (CREB), and the phosphorylation of myosin-related kinase B (TrkB). Thus, RSH is able to modulate anti-oxidant and neurotrophic pathways to exert memory improvement effects [88].

#### 3.3.2. Marine Invertebrates

A marine cyclic lipopeptide, Kahalalide F, isolated from a marine mollusk (Elysia rufescens), was studied to analyze its resistance to thermal or oxidative stress associated with lifespan extension in a nematode model [89]. Kahalide F significantly increased the expression of NLG-1, a neuronal protein induced by the mitochondrial stress expression of NLG-1, a neuronal protein [90]. Ultimately, Kahalide F significantly prolonged the lifespan and health span of nematode animals. Thus, the marine cyclic lipopeptide Kahalalide F can affect the lifespan and aging of nematode animals [15].

Sea cucumber is considered to be a food or drug source that can delay aging, enhance memory, and eliminate fatigue [53,91]. Additionally, then, three novel peptides, TP-WW-620 (FETLMPLWGNK), TP-WW-621 (HEPFYGNEGALR), and TP-WW-623 (KMYPVPLN), obtained from the protein hydrolysate of sea cucumber (Apostichopus japonicu), were investigated, which exhibited anti-oxidant activity and attenuated oxidative stress-induced mitosis, restored mitochondrial autophagy, reduced apoptosis, and consequently improved the survival of nematodes, and these three marine peptides exhibit potential value in extending lifespan and delaying aging [91]. In addition, two low hydrolysis sea cucumber peptides (SCP-1) and high hydrolysis sea cucumber peptides (SCP-2) obtained from sea cucumber protein hydrolysates were analyzed for their anti-fatigue effects, which were realized in male ICR mice with improved exercise capacity and reduced metabolite accumulation and muscle damage, as well as increased storage of muscle glycogen after the oral administration of the two peptides, respectively. Further analysis suggested that sea cucumber-derived SCP-1 and SCP-2 were able to inhibit oxidative stress and improve mitochondrial function by modulating NRF2 and AMPK signaling pathways, which contribute to the alleviation of physical fatigue [92].

#### 3.3.3. Marine Plants

**Figure 2:**
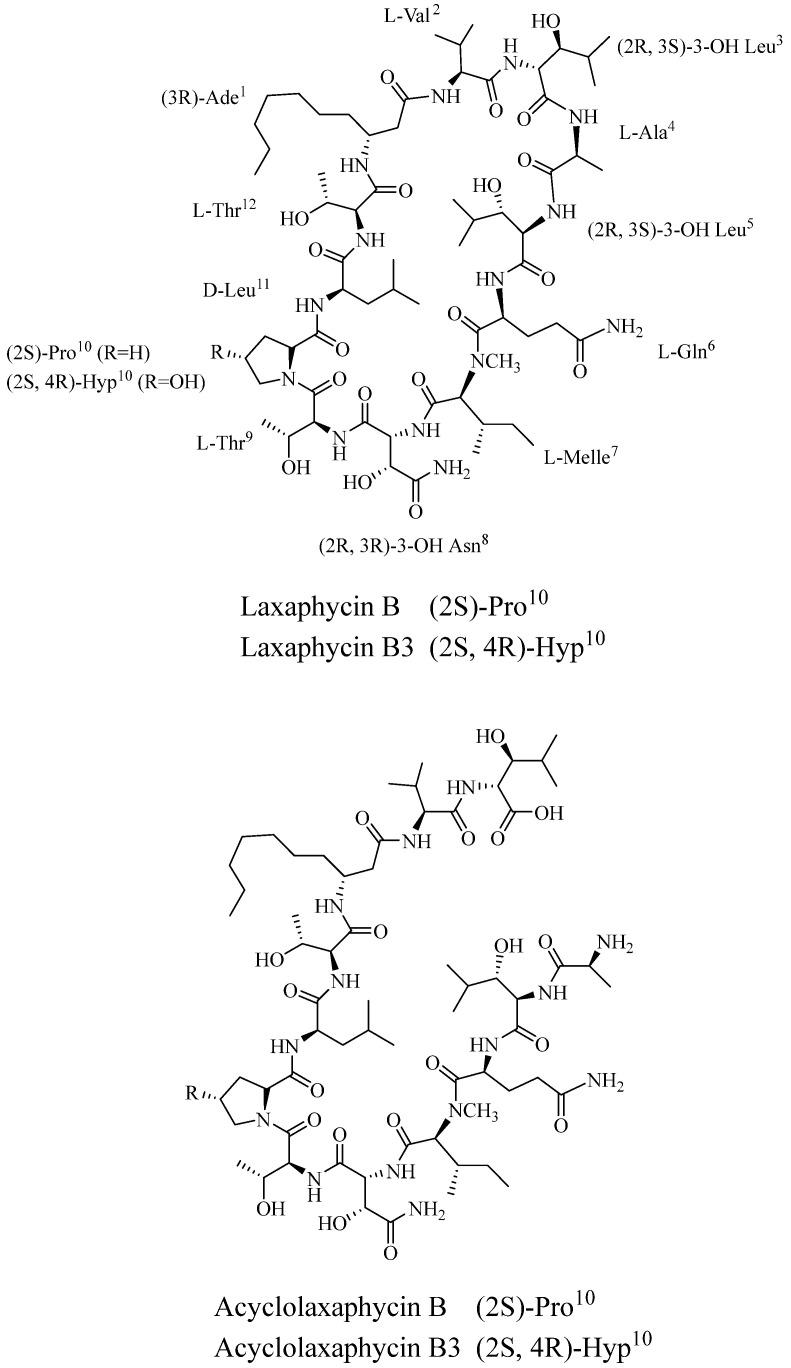
Chemical structures of Laxaphycins (laxaphycin B and acyclolaxaphycin B: R = H; laxaphycin B3 and acyclolaxaphycin B3: R = OH).

Besides the mitochondrial functional peptides of marine animal origin, researchers also isolated the non-ribosomal lipopeptides Bacillus toxins B and B3 and the non-cyclic Bacillus toxins B and B3 from the marine plant cyanobacterium Torabaena torulosa (Figure 2). The biological activities of these six novel peptides were then measured in SH-SY5Y human neuroblastoma cells, and it was found that B. subtilis toxins B and B3 could induce apoptosis and exert cytotoxic effects, whereas non-cyclic B. subtilis toxins were not cytotoxic; yet they were able to affect mitochondrial function and lead to a decrease in ATP levels. Analyses further revealed that B. subtilis toxins are able to promote AMPK phosphorylation and inhibit mTOR, thus affecting the autophagic process [93].

### 3.4. Immunomodulatory Peptides

During aging, cytokines produced by monocytes and macrophages are altered, with changes manifesting as reduced phagocytosis and decreased TLR expression [94], which ultimately alters the balance of the immune environment of the organism, resulting in immune dysregulation. In recent years, proteins, peptides, and protein hydrolysates of marine origin have been found to have a variety of activities by virtue of their anti-viral, anti-bacterial, and anti-fungal properties [29]. They play an increasingly important role in enhancing immune function, improving immunity [95], and alleviating the weakening of the immune system. Considering the relationship between the immune microenvironment and aging as a physiological process, the analysis of biopeptides with immunomodulatory functions in anti-aging is a valuable therapeutic prospect for aging and its related diseases. Therefore, certain current biopeptides of marine biological origin that exhibit promising therapeutic effects in immunomodulation are discussed below, and further studies are expected to analyze their potential value in the field of aging in the future. A summary can be found in Table 4.

#### 3.4.1. Marine Vertebrates

A well-studied active marine peptide with powerful immunomodulatory properties is fish protein hydrolysate (FPC), a valuable and little exploited source of potentially active biopeptides as an immunomodulatory food with the ability to enhance non-specific host defense mechanisms. As an example, firstly, in a study assessing the effect of FPC on mucosal immune responses in a mouse model, mice given FPC for 7 consecutive days showed enhanced phagocytic activity of peritoneal macrophages and increased numbers of IgA+ cells, IL-4, IL-6, and IL-10 in the lamina propria of the small intestine, as well as increased numbers of certain pro-inflammatory cytokines, such as IFNg and TNFa, maintaining intestinal homeostasis [95]. Another study on carp egg protein hydrolysates (CEPHs) found that CEPHs modulate splenic lymphocytes, NK cells, T cell subsets (CD4+ and CD8+), mucosal immunity (S-IgA), and serum immunoglobulin (IgA) in mice, enabling enhanced immune function in mice [96]. Additionally, in a study analyzing the effect of the enzymatic digestion of tuna cooking drip (CTD) (EH-TCD) on the immune function of Bal b/c mice, the use of TCD and EHTCD significantly increased the production of immunostimulatory cytokines (interleukin 10 and interleukin 2) and increased serum IgG1 and IgG2a levels, and even EH-TCD displayed an immune-enhancing effect stronger than TCD. These results suggest that EH-TCD and TCD might act as immunostimulatory agents to exert immune enhancing effects [97]. In a later study, after a low molecular weight peptide-NJP (<1 kDa) was obtained from Japanese yellow goby (Nibea japonica) protein hydrolysate, the evaluation of the immunomodulatory effects of this peptide on RAW264.7 cells revealed that NJP was able to promote cell proliferation by affecting cell cycle progression, and it significantly promoted cell phagocytosis, as well as pro-inflammatory cytokine secretion, and promoted IKK α/β by upregulating the protein expression degradation of IκB-α and the activation and translocation of p65 and nuclear factor NF-κB, as well as increasing nitric oxide (NO) synthesis. In conclusion, NJP is able to exert immunomodulatory effects through the NF-κB signaling pathway and affect cell viability [98].

Unfortunately, the effects of these peptides with immunomodulatory functions that influence the lifespan or aging-related characteristics of animals have not been sufficiently verified; however, the regulation of the key signaling pathways and their molecular expression by these immunomodulatory peptides in immunomodulatory functions may indicate the potential value of these peptides in anti-aging, which deserves to be studied and exploited in depth.

#### 3.4.2. Marine Invertebrates

A low molecular weight peptide (SCHPs-F1) was obtained from the head of red shrimp (Solenocera crassicornis), and SCHPs-F1 was found to exhibit dose-dependent ameliorative recovery from CTX-induced hepatotoxicity in mice by virtue of regulating the dual signaling pathways of Nrf2/anti-oxidant and NF-κB/inflammatory factors, finally reducing oxidative stress and inflammation in mice [99].

### 3.5. Intestinal Stabilizing Peptides

With increasing age, the microbial diversity in the gut microbiota [100] decreases in older age groups compared to younger age groups, with an increase in pathogenic bacteria and a decrease in probiotic bacteria [101]. Meanwhile, numerous studies on animals and humans have shown that the composition of the gut microbiota changes with the age of the host and that the gut microbiota is becoming a key factor in the aging process [102]. Whereas the use of anti-bacterial drugs or anti-biotic-like drugs is likely to have adverse consequences or resistance. Therefore, the development of gut probiotic peptides that are harmless and non-resistant to humans and the use of probiotic peptides to inhibit or activate pathogenic or probiotic bacteria to achieve the maintenance of biological gut microbial homeostasis to influence biological processes related to aging is a feasible option. However, research in this area of developing the use of marine biopeptides to modulate the gut microbiota in order to influence lifespan is currently limited, and work is mainly focused on fish protein hydrolysates; the specific research status is summarized in Table 5.

#### 3.5.1. Marine Vertebrates

It has been reported in the literature that two strains, LF3-1 and LF3-2, were isolated from tilapia, of which LF3-1 showed the significant inhibition of S. aureus and LF3-2 showed the significant inhibition of E. coli, while experimental speculation revealed that these two strains exerted their inhibition mainly by virtue of their lactic acid bacterial peptides, demonstrating the potential of lactic acid bacterial peptides of marine fish origin in maintaining intestinal microbial homeostasis. In addition, other researchers investigated and analyzed the effects of five different fish protein hydrolysate (HYD) diets in the growth of cod (Dicentrarchus labrax) and in the gut microbiota, comparing the results of body weight changes and survival rates and found that in terms of survival rates, the those of cod on the HYD1 diet were lower, while the survival rates of all other groups were within the normal range; regarding body weight changes, HYD1 and HYD2 had the lowest mean body weight, HYD4 and HYD5 had the highest mean body weight, and HYD3 was in the middle. The gut microbial community diversity of cod was then assessed, and it was found that the active microbiota profiles were dissimilar between the groups, with partially distinct differential colonization, so it was hypothesized that HYD significantly influenced the interaction between microbiota and cod larvae and thus the growth survival of cod, but the mechanism behind this effect is not clear [103].

#### 3.5.2. Marine Invertebrates

The phagocytosis of macrophages is one of the most important non-specific immune responses in the body and is essential for the uptake and degradation of infectious agents and senescent cells [104], it can be seen that the measurement of the phagocytic index (phagocytic rate) and phagocytic activity can be used to study the effect of substances on macrophage phagocytosis. Therefore, in an experiment using oyster peptides produced by protease hydrolysis to evaluate the effect of an enteral nutrition formula based on this peptide (OPENF) on primary immune function in mice, it was found that phagocytosis, such as splenic lymphocyte proliferation and natural killer (NK) cell activity, was increased in Bal b/c mice supplemented with OPENF. In addition, OPENF promoted intestinal absorption, improved food utilization, and maintained normal physiological functions in mice. These results suggest that OPENF has a stabilizing effect on the intestinal tract by enhancing the immune function of mice mainly through immunostimulatory effects, which in turn regulates intestinal absorption and metabolism and restores normal physiological functions in mice [105].

## 4. Other Features Associated with Aging

### 4.1. Neuroprotective Peptides

It is clear to us that aging is associated with physical degeneration leading to an increased risk of disease and death [106] and that among the different age-related diseases, the greatest impact of aging is related to neurodegenerative diseases, including Alzheimer’s disease (AD), Parkinson’s disease (PD), Huntington’s disease (HD), and so on. This is because neurodegenerative diseases are typified by mitochondrial dysfunction and the reactive production of oxygen species (ROS) [107] and because neurodegeneration and associated cognitive decline have a significant impact on health [108]. We have learned about the molecular mechanisms by which aging occurs from several aging theories, and similarly, at the core of most neurodegenerative diseases, rests the accumulation of abnormal proteins in the central nervous system (CNS), resulting in neuronal degeneration [109] and the natural loss of memory that occurs as a natural consequence of the concomitant aging and is thus consistent with multiple theories of aging. Neurodegenerative diseases are multifactorial diseases with complex mechanisms due to multiple causative events [110], of which oxidative stress and mitochondrial damage are major factors. On the one hand, the brain contains a large number of peroxide-prone adipocytes and high oxygen demand, making it particularly vulnerable to ROS [111]. ROS plays a key role in the biological evolution of nerve cell degeneration; ultimately, excess ROS can lead to neuronal damage and eventual memory and cognitive impairment through lipid peroxidation and protein alterations [112]. On the other hand, mitochondrial metabolism serves as a major source of high-energy intermediates and free radicals, and genetic or acquired mitochondrial defects may be responsible for neuronal degeneration [113]. Thus, there is a circular relationship between oxidative stress and mitochondrial dysfunction, and these molecular events that occur in the neural sources of the brain lead to neuronal cell death [114]. In conclusion, features of neurodegenerative diseases, including oxidative damage, mitochondrial injury, impaired neurotrophic signaling and neuronal death, are related to each other [72]. Therefore, searching for anti-oxidants and mitochondrial protectors as neuroprotective peptides to improve aging-related memory deficits and prevent and treat neurodegenerative diseases would be a very feasible option. Of course, some of the marine peptides that play roles such as anti-oxidants and mitochondrial protection have been described above, and some of the biofunctional peptides with neuroprotective effects caused by impacting neurotrophic signaling or neuronal activity identified by current research are described below. Interestingly, most of these functional peptides also have some association with evolutionary pathways influenced by oxidative and mitochondrial pathways.

As readers may already be aware, the hippocampus has many medicinal and health benefits for humans, including anti-aging, anti-fatigue, appetite enhancement, the treatment of pregnant women, and enhanced renal and neuroprotective effects [115]. In the first study on the optimization, isolation, and characterization of the hippocampus trimaculatus peptide (HTP), HTP-1(Gly-Thr-Glu-Asp-Glu-Leu-Asp-Lys (906.4 Da)) was found to be neuroprotective against amyloid-B42-induced PC12 cells by inducing the expression of the anti-apoptotic gene Bcl-2, which protects cells from neuronal death, and the upregulation of the pro-survival gene (Bcl-2) was found to exert a neuroprotective effect by increasing cell survival [116].

Furthermore, in studies of the use of animal venom against neurodegeneration, it has been found that animal venom contains natural peptides that play a role in neuroprotection [117]. For example, an in vivo study using a rat spinal cord ischemia model confirmed that MVIIC, one of ω-conotoxins from the venom of the fish-eating marine snail *Conus magus* and a new peptide targeted to presynaptic Ca^2+^ channels [118], reduced glutamate release and calcium ion influx into cells, preserved neuronal integrity, reduced cell death and hemorrhage, and improved performance in behavioral tests [119].

In another experiment, female C57BL/6J mice were fed with diets containing marine collagen peptide (MCP) obtained from the skin enzymatic digestion of salmon (Oncorhynchus Keta), and the mice were found to have significantly better passive avoidance, spatial memory, and learning abilities in the model test group than in the older control group, and the learning memory abilities of the mice were not significantly different from those that were younger. The analysis showed that MCP attenuated oxidative stress, reduced neuronal apoptosis, and upregulated the expression of the brain-derived neurotrophic factor (BDNF) and postsynaptic density protein 95 (PSD95), showing neuroprotective effects [120]. There is also a study on the neuroprotective effect of marine collagen peptides (MCPS) obtained from the enzymatic digestion of salmon skin on male rats in perinatal asphyxia (PA), where it was found that intervention with different doses of MCPs did not improve the poor behavior of PA rats in a short period of time, but the number of hippocampal neurons and brain cells increased and the behavioral performance of the rats accompanied by the prolonged use of MCPs improved. In addition, the data showed a significant increase in the expression of p-CREB and BNDF in the hippocampus, all of which again suggest a role for MCPs in neuroprotection [121].

In addition, protein hydrolysate (BPH) from a deep-sea bycatch, Benthosema pterotum, was found to have anti-oxidant activity, activating the intracellular anti-oxidant system and significantly reducing H_2_O_2_-induced reactive oxygen species (ROS) and apoptosis in human neuroblastoma SH-SY5Y cells. It also improved the expression of D -galactose (D-gal)-induced memory and learning deficits in neurodegenerative/aging ICR mice, alleviating aging and/or age-related neurodegenerative diseases, and exhibiting neuroprotective effects [122].

### 4.2. Anti-Photoaging Peptides

As we age, our bodies undergo many changes and our skin is no exception. Natural and environmental factors cause the aging of human skin [123]. The main source of skin aging is ultraviolet radiation (UVR), which causes skin cells to produce harmful reactive oxygen species (ROS). Currently, anti-skin aging is mainly achieved by using various pharmaceutical formulations with anti-oxidants in medicinal products to inhibit ROS and achieve photoprotection [124].

An increasing number of novel molecules from marine flora and fauna now exhibit potent and effective dermatological activities, such as secondary metabolites isolated from macroalgae, including carotenoids and polyphenols, which have shown anti-oxidant, anti-aging, and anti-inflammatory activities [125]. Thus, using the biopeptides of marine origin as research subjects, numerous marine bioprotein hydrolysates have been found to possess anti-oxidant and tissue regeneration properties as well as anti-bacterial and matrix metalloproteinase inhibiting activities, thus displaying efficient anti-photoaging activities for application in the development of skin anti-aging cosmeceuticals [20].

On the one hand, we know that fish by-products, such as skin, bone, head, and scales, are rich in collagen with multiple biological activities [126]. Therefore, their conversion into advanced products through biological methods is a very valuable way to recycle them. On the other hand, fish skin is abundant in gelatin and collagen, which can be hydrolyzed to produce bioactive peptides of 2–20 amino acid sequences. Current studies have shown that peptides purified from fish skin possess a range of biological activities, such as anti-hypertensive, anti-oxidant, anti-microbial, neuroprotective, anti-hyperglycemic, and anti-aging [127]. First, the anti-photoaging effect of LSGYGP, a peptide purified from tilapia (Oreochromis niloticus) skin, was evaluated. It was found that this peptide improved the skin condition of UV-irradiation-induced photoaging in mice using its anti-oxidant activity [128]. Next, two peptides with different sequences, M1 (GEIGPSGGRGKPGKDGDAGPK) and M2 (GFSGLDGAKGD), were identified from cod skin gelatin hydrolysate (CGH). Of these, the first sequence of peptides down-regulated MMP-1, p-ERK, and p-p38, while the second sequence of peptides down-regulated p-JNK in the MAPK signaling pathway to achieve anti-photoaging activity [129]. In addition to fish skin-derived anti-aging peptides, other waste, such as fish bones, fish scales, and digestive organs [130], which are utilized to obtain collagen hydrolysates, may also contribute to improving skin aging [131].

A sea star organism, Asterias pectinifera, could be an eco-friendly source of non-toxic and highly water-soluble low molecular weight collagen peptides with good efficacy for wound-healing, bone regeneration, and skin protection [132]. Thus, in an experiment investigating the use of the elastic nanoliposomes of collagen peptides from starfish for cosmetic applications, the elastic nanoliposomes of low molecular weight collagen peptides derived from Asterias pectinifera were found to reduce the expression of MMP-1 induced by UV radiation photoaging and exhibit anti-photoaging effects [133].

In addition, a recent study found that a variety of peptide active ingredients, Ops, could be isolated from enzymatic hydrolysate (OAH) of Oyster Konjac (Crassostrea Hongkongensis). Ops-pretreated HaCaT cells exposed to UV irradiation were assayed and, it was found that these small peptides inhibited MMP-1 by anti-oxidants, promoting cell proliferation, inhibiting β-galactosidase, alleviating cell senescence, and inhibiting MMP-1 expression, ultimately exhibiting anti-photoaging activity [134].

### 4.3. Angiotensin I-Converting Enzyme (ACE) Inhibitory Peptides

In nematodes, the relationship between ACE inhibition and lifespan was explored using the acn-1 gene, a homolog of nematode ACE [135]. Angiotensin-converting enzyme inhibitors reduce acn-1 activity, which in turn prolongs nematode lifespan, enhances stress resistance, and delays age-related degenerative changes as measured by pharyngeal pumps. Furthermore, ACE inhibition was also found to be associated with prolonged lifespan using Drosophila [136] and mice [137] as experimental animals. Therefore, this review summarizes and outlines current peptide substances of marine origin that are able to demonstrate anti-aging potential through the inhibition of angiotensin I-converting enzyme (ACE) activity.

At first, an oligopeptide with 11 amino acids (VECYGPNRPQF) and other smaller peptides, such as VVPPA and IVVE, were isolated from Chlorella vulgaris, then many tripeptides or tetrapeptides were obtained by the pepsin digestion of Spirulina platensis, and the IC_50_ values of these small peptides for ACE were further analyzed. It was found that they all inhibited ACE and could be candidates for skin anti-aging [138]. In addition, ACE inhibition studies were performed on dipeptides produced by different enzymatic digestions of another alga, Undaria pinnatifida, such as VW, IW, and LW, which were administered alone or orally to dipeptides of the above origin in spontaneously hypertensive rats (SHR) in order to study the in vitro angiotensin-converting enzyme inhibitory activity and in vivo hypotensive effects in rats. It was found that the small peptide obtained by the hydrolysis of wakame had strong ACE inhibitory activity, the systolic blood pressure of SHR was significantly reduced, and the increase in systolic blood pressure was significantly inhibited [139,140]. Alaska pollock (*Theragra chalcogramma*) was also found to produce dipeptides as well as tripeptides, of which the tripeptides [141] were isolated from fish skin and oligopeptides (FGSTRGA) were isolated from the framework hydrolyzed proteins [142], which were identified as having ACE inhibitory activity. Then, when studying the anti-oxidant properties and angiotensin converting enzyme (ACE) inhibitory activity of zebrafish (*Salaria basilisca*) protein hydrolysates (ZBPHs) in tetrazolopyrimidine diabetic rats (AIDR), it was found that the gavage of different ZBPHs to AIDR rats showed a significant decrease in anti-oxidant enzyme activity and a significant increase in malondialdehyde (MDA) levels in the liver tissue, and that ZBPHs could modulate ACE activity and prevent diabetes-related complications [143]. In addition, peptides from pearl oysters and clams [144,145] as well as shrimp peptides [146] were also found to have ACE inhibitory effects. In another study on the effect of mudskipper (Misgurnus Anguillicaudatus) peptide in spontaneously hypertensive rats, the mudskipper peptide increased the activity of SOD, CAT, and GPx and minimized their systolic blood pressure, indicating its angiotensin-converting enzyme (ACE) inhibitory activity [147]. Furthermore, sardinella (*Sardinella aurita*) protein hydrolysates (SPHs) exhibited good hypolipidemic properties and anti-oxidant activity in rats, a model of hyperlipidemia. By feeding a cholesterol-rich SPHs diet to rats, SPHs were found to reduce the activity of serum enzymes, such as LDH; reduce liver damage; and increase anti-oxidant enzyme activity, showing better hypolipidemic and anti-oxidant activities [148]. Finally, in a study concerning fish skin hydrolysates possessing abundant proteins, two peptides MVGSAPGVL and LGPLGHQ from skate (*Okamejei kenojei*) skin were found to have protective effects on human endothelial cells with angiotensin II-induced endothelial dysfunction. Results revealed that both peptides protect human endothelial cells by the activating PI3K/Akt-dependent signaling pathway in human endothelial cells and show vasodilatory effects. NF-κB and MAPK signaling pathway activation are involved in the regulation of inflammatory mediators (IL-6, VCAM, and ICAM); therefore, skate skin peptides may be used for the prevention of aging-related diseases, such as cardiovascular disease, by inhibiting ACE activity [149].

### 4.4. Other Functional Peptides

We know that in addition to the relatively common aging-related diseases, such as neurodegeneration, skin aging, and hypertension, as described above, there are also more general symptoms associated with aging, such as obesity and the accompanying high blood sugar or fat levels, fatigue, and an increased incidence of cancer. There are also certain peptides of marine origin that do not clearly show anti-aging effects but play a role in these aging-related aspects, as described below.

Protein hydrolysates from yellow-winged whales have been found to have good anti-oxidant activity and are used in the food industry as natural additives with anti-oxidant properties [150]. It has also been shown that protein hydrolysates (GPHs) from goby, particularly GPHTF, promote natural defenses against ROS generated by high cholesterol diet-induced hyperglycemia and are well-behaved anti-oxidant peptides with ant glycemic effects [151]. In addition, the peptide component (SBP-III-3) in the protein hydrolysate of the rhubarb swim bladder was found to produce anti-fatigue effects in mice by inhibiting oxidative responses, including DNA damage [152].

### 4.5. Multifunctional Peptides 

As we have already discussed, aging is the result of multiple factors, and marine peptides that only target one mechanism of aging may have a certain effect on one part of the metabolic pathway of aging, but the resulting therapeutic effect is not very satisfactory. Therefore, with reference to the synergistic therapeutic measures that work well in disease treatment, we have identified certain marine peptides that intervene in aging-related processes through multiple targets and multiple pathways, and these can affect multiple targets to produce interactive results [153]. These multi-targeted peptides are undoubtedly valuable reference objects for future research and the development of anti-aging marine peptide drugs or drug candidates. Taking multicellular signaling pathways and complex networks as an entry point, we will focus on the multiple important pathways through which active marine peptides regulate aging interactions in order to achieve possible healthy lifespan extensions. 

In the study of in vitro cellular bioactivities of gelatin hydrolysate (GH) obtained from the enzymatic digestion of the unicorn leatherjacket (Aluterus monoceros), GH protected against DNA damage and decreased anti-oxidant enzyme activity under oxidative stress and also showed immunomodulatory and anti-proliferative potential in colon cancer (Caco-2) cells. GH shows biological activity in several ways and is a peptide compound with anti-aging medicinal value [154]. In a separate study, the gelatin hydrolysates of fish skin from other fish species exhibited anti-oxidant, immunomodulatory, and anti-proliferative effects. The study of gelatin hydrolysates from seabass (Lates calcarifer) revealed that the substance exhibited multiple biological activities similar to the previous hydrolysate (CH), in addition to exhibiting the inhibition of HepG2 cells, again demonstrating the value of fish skin hydrolysates for development as anti-aging agents [155].

Furthermore, two multifunctional peptides, YSQLENEFDR (Tyr-Ser-Gln-Leu-Glu-Glu-Asn-Glu-Phe-Asp-Arg) and YIAEDAER (Tyr-Ile-Ala-Glu-Asp-Ala-Glu-Arg), were isolated and purified from the meat and viscera extracts of Neptunea arthritica cumingii (NAC), both of which were found to possess high anti-oxidant, angiotensin-converting enzyme (ACE) inhibitory and anti-diabetic activities and protect skin cells from oxidative damage, indicating their potential application in the prevention of aging-related diseases [156]. A novel peptide, KVEPQDPSEW (AATP), was isolated from abalone (Haliotis discus hannai), and it was found that AATP was not only able to block MAPKs and NF-κB pathway to downregulate the matrix metalloproteinase MMPs but also block AKT/mTOR signaling to inhibit pro-angiogenic factor (VEGF) and ultimately inhibit HT1080 cell metastasis and VM formation. The anti-metastatic and anti-vascular effects of AATP on HT1080 cells exhibit its potential anti-tumor activity, but the aging-related signaling factors and pathways it acts on (e.g., MMPs and mTOR) also exhibit its possible anti-aging activity, which is a prompt for in-depth studies and further validation [157,158].

When it comes to important marine biopeptides with multiple functional activities, such as anti-oxidant, anti-hypertensive, anti-wrinkle, anti-fatigue, and so on [159], there is no doubt that oyster peptides (OPs) are more frequently studied. In addition to the anti-oxidant oyster peptides discussed above, some other oyster peptides that are closely associated with the onset and outcome of aging are presented below. For example, a study on the enhancement of the in vitro anti-oxidant activity and in vivo anti-fatigue activity of oyster peptides revealed that OPs are able to scavenge free radicals and inhibit lipid peroxidation in order to exert anti-oxidant activity on the one hand, while on the other hand, they are able to prolong the time from swimming to exhaustion to exert anti-fatigue effect in mice, which provides an essential basis for the development of OPs as anti-oxidants and anti-fatigue compounds for anti-aging drugs [160]. In another study on the anti-wrinkle activity of oyster peptides, it revealed that the hydrolysate (OH) of oyster (Crassostrea Gias) was able to regulate the expression of MAPKs, AP-1, MMPs, and transforming growth factor β receptor II as well as the phosphorylation of Smad3 in order to affect collagen breakdown and production, producing anti-wrinkle effects, in addition to its anti-cancer properties, which may correlate with its anti-oxidant effects [161]. 

These marine peptides, which we discussed above, have multiple biological activities due to their action on several metabolic pathways related to the onset of aging and aging-related diseases, and although the available studies do not directly show their intervention in lifespan extension, the interaction between these effects indirectly demonstrates the anti-aging effect of these multifunctional peptides.

## 5. Partial Peptide Analysis in Silico

Traditional methods for peptide screening and drug design are often time-consuming, costly, and labor-intensive. In order to predict peptides with different biological activities, over the past two decades, a number of in silico tools have been developed for the design of peptide-based drugs. These tools have proven to be catalysts for peptide screening and drug design [162]. Therefore, more and more researchers have focused on the integration of bioinformatics tools for the healthcare industry, which can be used to predict potentially bioactive peptides [163].

Through the in silico synthesis and molecular docking simulation of bioactive peptides obtained from Sargassum Maclurei protein hydrolysis, a new peptide RWDISQPY was found to bind to the active sites S1 and S2 of ACE through a short hydrogen bond, which had a strong ACE inhibition ability (IC_50_: 72.24 μM) and decreased systolic and diastolic blood pressure in spontaneously hypertensive rats at 150 mg/kg (*p* < 0.05) [164]. In one study, the in silico analysis of hydrolysates from the chloroplast proteins of Red Alga Grateloupia asiatica and the comparison of their iSD_50_ values with IC_50_ values in vitro revealed that hydrolysates from WSP possessed numerous ACE-inhibitory peptides [165]. These results suggested that marine peptides could be developed into functional anti-hypertensive and anti-aging drugs.

In a study that aimed to predict the in vivo function of peptides produced from the Atlantic sea cucumber through in vitro anti-oxidant activity, peptide sequence analysis, and in silico analysis, sea cucumber peptides were found to have potential myeloperoxidase (MPO) inhibitory activity, predicting the ability to inhibit oxidative stress in vivo [166]. In a bioinformatics study on the proteome of laver (*Porphyra haitanensis*), bioactive pancrease-digested peptides with anti-oxidant activity were identified by searching a library. The anti-oxidant ability of laver extracts was confirmed by various in vitro experiments, indicating the potential of laver as natural anti-oxidants for health care and disease treatment [167]. The in vitro and silico analysis of the potential anti-oxidant activity of the viscera of sea snails (*Turbo cornutus*), nine bioactive peptides with hydrogen peroxide scavenging activity were detected in the protein substances composed of the snails. It showed protective effect on oxidative stress of HepG2 cells treated with hydrogen peroxide and binding inhibitory effect on MPO. These results suggest that the viscera of sea snail (*Turbo cornutus*)-derived active peptides can be used as functional pharmaceutical components for human health due to their oxidation properties [168]. In an experiment that predicted the biological activity of skin collagen peptides by combining them with silicon in vitro, the prediction of the biological activity and characteristics of polypeptides (PPA, PPN, PPS, PBA, PBN, and PBS) obtained by the ultrafiltration of collagen hydrolysate of bigeye tuna (*Thunnus obesus*) skin collagen were studied by silicon analysis [169]. It was found that all of these had anti-oxidant activity [169]. This indicates that these peptides are potential compounds for pharmaceutical and health applications.

Interestingly, the in silico analysis of the peptide profile data of active peptides produced by the enzymatic digestion of isolated proteins from sea cucumber by-products, such as flowers and offal, which are often disposed of as waste, revealed that sea cucumber-derived peptides may have the potential to inhibit lipid peroxidation processes and exert anti-oxidant activity [170].

## 6. Conclusions and Future Perspective

This review takes the current popular five major aging theories as its background; starts from different mechanisms of action; analyzes the aging intervention effects at different levels, according to the main manifestation characteristics of aging and its key pathways; and finally, mainly by classifying biological species, summarizes and outlines the marine origin peptide compounds with lifespan-extending or aging-delaying effects reported in the current literature, as well as discussing the development prospects of marine origin bioactive peptides as anti-aging drugs. 

In contrast to traditional terrestrial sources and small molecule chemosynthetic drug development, the collection of studies presented in this review focuses on the reliable sources, action characteristics, technical methods, and results of potential anti-aging peptides in this research area of marine organisms, highlighting the importance of the ocean as a rich source of drugs and the frontiers of peptides in drug development. At the same time, the mechanism of action and characteristics of these marine peptides are presented, especially at the molecular, cellular, and systemic levels, as opposed to the single level of action. Considering other related chronic diseases that may be caused by aging and the existence of multi-mechanism synergism, we also separately introduced certain special multifunctional peptides with “multi-target, multi-pathway, and multi-action” properties. In addition, in contrast to the current homogeneity and generalization of marine species, this paper not only discusses marine animals and plants but also clearly introduces current aging studies at the marine organism level by classifying these organisms in a more detailed way, which is of special significance for researchers aiming to develop different marine organisms in the future. Finally, in addition to discussing the current results of the action of these marine peptides in biochemical in vivo and ex vivo experiments, this review also combines certain findings from the computer-aided drug design panel, such as in silico analysis, which is currently very popular in the field of drug development, to not only enrich the data of marine anti-aging peptide compounds but also to point out more advanced research tools for the future pharmaceutical industry.

However, in terms of developmental purposes, our current research on aging tends to be fragmented and singular, and aging is a process that occurs at the level of the organism as a whole, so there is uncertainty regarding the experimental validation of a particular mechanism, and there are other mechanisms of action for aging besides the five prevalent theories we discussed above. At the same time, the characteristics that we currently rely on to determine whether aging is occurring are diverse, and thus, studies aiming to slow down one or several aging characteristics are superficial, suggesting that multiple pathways of action may be needed to truly delay aging. Moreover, from the viewpoint of research objects, the number and species of marine organisms are very large. Additionally, it is very difficult to find specialized organisms suitable for drug research and development, while a large number of studies on active marine peptides are mainly focused on analyzing collagen obtained from fish skin hydrolysates for skin anti-aging in pharmaceutical products, while drug development for other organisms in the intervention of aging is very limited. Additionally, in terms of obtaining marine active peptide materials, a considerable number of marine peptides are almost always obtained by enzymatic or chemical hydrolysis, which has the disadvantage of being high cost and low yield. These problems cause some obstacles in assessing the functional value of marine peptides and the commercial applications of peptides. Furthermore, the current evaluation of the function of anti-aging drugs often relies on animal experiments, while few peptide drugs are actually used in human clinical trials.

Therefore, given the advantages and disadvantages of studying and developing marine peptide-based anti-aging drugs, finding operational methods to effectively assess biological aging as measured by specific aging markers and multiple pathways to elucidate the mode of action of aquatic peptides, discovering low-cost and high-yield peptide extraction techniques, expanding the experimental population for research, breaking the singularity of marine peptide uses, and aiding the use of more efficient in silico analytical tools to deeply develop these undervalued marine peptides are important areas for the pharmaceutical industry to develop in the future in terms of anti-aging drugs.

## Figures and Tables

**Table 1:** Anti-oxidant active peptides.

Source		Peptides	Outcomes	Activities	Ref.
**Marine Vertebrates**					
Chum Salmon (Oncorhynchus keta) skin		MCPs	inhibit the decrease in the activities of anti-oxidant enzymes and in the levels of lipid peroxidation; increased the mean lifespan; decreased overall spontaneous tumor incidence	anti-oxidant, anti-tumor	[46]
Defatted round scad (Decapterus maruadsi)		P1, P2, P3, P4, P5, P6, P7, P8	longer lifespan; higher survival rate; higher superoxide dismutase (SOD) and catalase (CAT) activities	anti-oxidant, anti-aging	[47]
Crimson snapper		CSSPs	prolong the mean lifespan; reduce the accumulation of peroxide products; improve the activity of anti-oxidant enzymes; up-regulate the expression of anti-oxidant-related genes	anti-oxidant, anti-aging	[48]
Atlantic cod (Gadus morhua)		SWP-I, SWP-II	scavenge DPPH•, HO•, and O^2−^•; high Fe^2+^-chelating activity; increase cell viability rates; suppress SA-β-gal activities; inhibit apoptosis rates in H_2_O_2_-induced premature senescent 2BS cell	anti-oxidation, anti-aging	[49]
Grass carp (Ctenopharyngodon arondo) skin		PTSPL, GPGPGL, VGGAP	exhibit high scavenging activity on DPPH radical, hydroxyl radical, and ABTS radical; inhibit the peroxidation in linoleic acid model system	anti-oxidation	[50]
**Marine Invertebrates**					
Jellyfish collagen		JCH	increase the climbing time; reduce blood lactic and BUN levels; increase hepatic glycogen and muscle glycogen; higher MDA contents of serum and liver homogenate; a decreased level in GSH-Px activity of serum and SOD activity of liver homogenate; alleviate fatigue in mice	anti-oxidation, anti-fatigue	[51]
Oyster		OH-I, OH-II	prolong swimming time; increase the content of muscle glycogen and liver glycogen; reduce the content of blood urea nitrogen (BUN)	anti-oxidation, anti-fatigue	[14]
Sea cucumber	(Cucumaria frondose)	CFH	improve learning memory and cognitive impairment	anti-oxidant	[56]
	(Stichopus variegates)	SVH-PF, SVH-CAH-PF	up-regulate Klotho expression, activating SOD and GSH-Px; inhibit lipid peroxidation and protein oxidation	anti-oxidation, anti-aging	[54]
	(Apostichopus japonicus)	AjPH(GF2, GF3)	increase the survival rate; reduce ROS level; delay physiological aging	anti-oxidant, anti-aging	[53]

**Table 2:** Peptides regulating hormone metabolism.

Source	Peptides	Outcomes	Activities	Ref.
**Marine Invertebrates**				
Mussel (Mytilus Edulis)	MCPs	attenuate the properties associated with cellular senescence; increase glutathione (GSH) level; recover mitochondrial transmembrane potential; improve the transcriptional activity of Prx1, NAMPT and SIRT1	anti-oxidant, anti-tumor	[64,65]
Purple sea urchin (Strongylocentrotus nudus)	SnP7, SnP10	reduce reactive oxygen species (ROS) level and the expression of superoxide dismutase-3 (SOD-3) and heat shock protein-16.2 (HSP-16.2) in oxidation-damaged nematodes; induce DAF-16 nuclear translocation and the expression of stress-related genes, such as sod-3	anti-oxidant	[66]
Clam (Meretrix meretrix)	MmP4, MmP11, MmP19	promote nuclear translocation of the DAF-16/FOXO transcription factor, a pivotal regulator of stress response and lifespan; induce the expression of superoxide dismutase 3 (SOD-3),	anti-oxidant	[67]
Arca subcrenata	D2-G1S-1, G2-G1S-2	exhibit potent DPPH ^•^ and ABTS ^•+^ radical scavenging activities and ferric-reducing anti-oxidant ability; enhance the mean lifespan of Caenorhabditis elegans and significantly improve age-associated physiological functions in the nematode; downregulate gene age-1 and upregulate stress-inducible genes	anti-oxidation, anti-aging	[68]
**Marine Vertebrates**				
Fish roe	FRP	promote insulin secretion and cell viability; reduce apoptosis and intracellular ROS levels; increase the activity and content of anti-oxidant-related enzymes	Anti-oxidant	[69]

**Table 3:** Mitochondrial functional peptides.

Source	Peptides	Outcomes	Activities	Ref
**Marine Vertebrates**				
Anchovy (Coilia mystus)	PYCS, CVGSY	alleviate memory impairment; suppression of ROS production and Ca^2+^ influx	oxidative stress, neuroprotection	[87]
Round scad (Decapterus Maruadsi)	RSH	reverse the cognition deficits induced by sleep deprivation; alleviate oxidative stress; up-regulate the expression of anti-oxidant defense-related proteins; improve the expression of brain-derived neurotrophic factor (BDNF), the phosphorylation of cAMP response element-binding (CREB) and tropomyosin-related kinase B (TrkB) in vivo	anti-oxidant	[88]
**Marine Invertebrates**				
Mollusk (Elysia rufescens)	Kahalalide F	increase the expression of NLG-1; extend the lifespan and health span of nematode	anti-aging	[87,89]
Sea cucumber (Apostichopus japonicu)	TP-WW-620, TP-WW-621, TP-WW-623	protect cells against hydrogen peroxide; reduce the oxidative stress induced through the depletion of cellular glutathione; decrease mitochondrial superoxide levels; alleviate mitophagy in human neuroblastoma cells	anti-oxidant	[91]
Sea cucumber	SCP-1, SCP-2	improve exercise performance in mice; reduce metabolism accumulation and muscle injury and enhanced glycogen storage in mice; inhibit oxidative stress and enhance energy metabolism in mice; modulate oxidative stress and mitochondria function-related protein expression in the skeletal muscles	anti-fatigue, oxidative stress, mitochondrial	[92]
**Marine plants**				
cyanobacteria (Anabaena torulosa)	Laxaphycins (laxaphycins B and B3, acyclolaxaphycins B and B3)	induce apoptosis; affect mitochondrial functioning; promote AMPK phosphorylation; inhibit mTOR	Cytotoxic, mitochondrial, pro-apoptotic, autophagic	[93]

**Table 4:** Immunomodulatory peptides.

Source	Peptides	Outcomes	Activities	Ref
**Marine Vertebrates**				
Fish	fish protein hydrolysate (FPC)	increase the number of IgA^+^ cells, IL-4, IL-6, and IL-10 in the lamina propria; increase certain pro-inflammatory cytokines; maintain the intestinal homoeostasis	immunomodulatory	[95]
Carp egg	carp egg protein hydrolysates (CEPHs)	enhance the proliferation of spleen lymphocytes; increase the splenic natural killer cell cytotoxicity, mucosal immunity (secretory immunoglobulin A) in the gut and level of serum immunoglobulin A; increase the percentages of CD4+ and CD8+ cells in spleen	immunomodulatory	[96]
Tuna cooking drip (CTD)	enzymatic digest of tuna cooking drip (EH-TCD)	increase the production of immunostimulatory cytokines (interleukin-10 and interleukin-2); increase serum IgG1 and IgG2a levels	immunomodulatory	[97]
Japanese yellow goby (Nibea japonica)	low molecular weight peptide (NJP)	promote cell proliferation and have no significant toxic effects on RAW264.7 cells; promote phagocytic capacity and the secretion of proinflammatory cytokines TNF-α, IL-6, and IL-1β; promote cell cycle progression and increase the percentage of cells in G0/G1 phase	immunomodulatory	[98]
**Marine Invertebrates**				
Red shrimp (Solenocera crassicornis)	SCHPs-F1	alleviate the CTX-induced hepatotoxicity; reduce the P450 protein content; ameliorate the pathological structural disorder of hepatic tissue in mice; reduce the MDA levels, and increase the activity of hepatic anti-oxidant enzymes; restore levels of proinflammatory factors; improve the CTX-induced hepatic oxidative stress; reduce the secretion of the pro-inflammatory cytokines	anti-oxidant, anti-inflammation	[99]

**Table 5:** Intestinal stabilizing peptides.

Source	Peptides	Outcomes	Activities	Ref
**Marine Vertebrates**				
Cod (Dicentrarchus labrax)	HYD, HYD2, HYD3, HYD4, HYD5	stimulate larval growth with HYD4 and HYD5; stimulate the relative activity of enzymes in the brush border membrane of enterocytes with HYD3 and HYD4	gut microbial	[103]
**Marine Invertebrates**				
Oyster	OP(ENF)	improve intestinal peristalsis hyperfunction and absorption of mice; interfere with humoral and cell-mediated immunity; enhance cell-mediated immunity in immunosuppressive mice; increase activity of phagocyte	immunostimulatory	[105]

## Data Availability

Not applicable.

## Supplementary Materials

The following supporting information can be downloaded at: https://www.mdpi.com/article/10.3390/md21030144/s1, Table S1: Amino acid composition of MCPs; Table S2: Amino acid sequences of AjPH; Table S3: Amino acid sequences of MP identified by HPLC-Q-TOF; Table S4: Molecular weight distribution and amino acid composition of RSH; Table S5: Amino Acid profile of carp roe hydrolysates produced by pepsin, trypsin, and Alcalase; Table S6: The amino acid composition of the low molecular weight peptides from byproduct shrimp head of Solenocera crassicornis; Table S7: Amino acid composition of MCP from salmon skin; Table S8: Amino acid composition of the MCPs from the skin of chum salmon; Table S9: Amino acid composition of the collagen peptides derived from Asterina pectinifera; Table S10: The amino acid composition of LP; Table S11: Amino acids composition (%) of UGP and GPHs.
