# Supplementary material for: Potential Active Marine Peptides as Anti-Aging Drugs or Drug Candidates
Source: Mar Drugs. 2023 Feb 23;21(3):144. doi: 10.3390/md21030144 (PMC10053682; doi:10.3390/md21030144)
Supplement: Supplementary file 1 [file marinedrugs-21-00144-s001.zip › marinedrugs-2202319-supplementary.pdf]

Supplementary Data

Potential marine active peptides as anti-aging drugs or drug candidates (2000-2022)

Hui Yang [1], Qi-Ting Zhang [2], Bin Zhang [3,*], Yu-Fen Zhao [1], Ning Wang [1,*]

1 Institute of Drug Discovery Technology, Ningbo University, Ningbo, 315211, Zhejiang, China

2 College of Chemistry and Chemical Engineering, Xiamen University, Xiamen, 361005, Fujian, China

3 Li Dak Sum Yip Yio Chin Kenneth Li Marine Biopharmaceutical Research Center, Department of Marine Pharmacy, College of Food and Pharmaceutical Sciences, Ningbo University, Ningbo, 315800, Zhejiang, China

* Correspondence: Bin Zhang, zhangbin1@nbu.edu.cn; Ning Wang, wangning2@nbu.edu.cn;

Table S1 [1]**.** Amino acid composition of MCPs

Amino acid	Number of residues/100 residues
**Glycine**	23.77
**Glutamic acid**	12.22
**Proline**	9.79
**Hydroxyproline**	7.51
**Aspartic acid**	7.29
**Alanine**	6.59
**Arginine**	6.08
**Lysine**	5.66
**Leucine**	4.64
**Serine**	4.23
**Valine**	2.94
**Isoleucine**	2.57
**Threonine**	2.53
**Phenylalanine**	2.51
**Histidine**	1.61
**Methionine**	0.03
**Tyrosine**	0.03

Table S2 [2]**.** Amino acid sequences of AjPH

A. japonicus protein hydrolyzate fractions	Sequence	No. of residues
**GF2**		
**M1**	TYPE	4
**M2**	FHLPI	5
**M3**	PIWL	4
**M4**	DYVNK	5
**M5**	VFESV	5
**M6**	PRPXL	5
**M7**	TDYEWL	6
**M8**	QTHPTY	6
**M9**	VTPLG	5
**M10**	IIKP	4
**M11**	RFDT	4
**GF3**		
**M1**	DYEW	4
**M2**	YPXPL	5
**M3**	HPTYL	5
**M4**	NFHLP	5
**M5**	VFESVXR	7

Table S3 [3]**.**Amino acid sequences of MP identified by HPLC-Q-TOF

**Number**	**Sequence**	**Number of residues**	**Molecular weight (Da)**
**1**	QIDEINVE	8	961.47
**2**	AITEVDLER	9	1046.56
**3**	LEDELLAEK	9	1060.56
**4**	EDSYEETIR	9	1142.51
**5**	LAITEVDLER	10	1159.64
**6**	IQLLEEDLER	10	1258.67
**7**	KLAITEVDLER	11	1288.74
**8**	IEELEEELEAER	12	1489.71

Table S4 [4]**.** Molecular weight distribution and amino acid composition of RSH

**Molecular weight**	**>10 kDa**	**5–10 kDa**	**3–5 kDa**	**1–3 kDa**	**<1 kDa**
**amino acid**	Contents of amino acids in RSH (g per 100 g)				
**Ile(Leu)**	3.76(6.33)				
**Ala**	4.97				
**Val**	4.19				
**Thr**	3.91				
**Ser**	3.94				
**Glu**	13.32				
**Gly**	4.76				
**Asp**	9.36				
**Phe**	3.36				
**Pro**	6.60				
**Tyr**	3.25				
**Asn**	ND				
**Lys**	7.92				
**Gln**	ND				
**Met**	2.57				
**His**	3.74				
**Arg**	11.67				
**Trp**	ND				
**Cys**	1.22				
**HAA**	36.56				
**PCAA**	23.34				
**AAA**	6.94				

Hydrophobic amino acids (HAA): Ala, Cys, Val, Met, Ile, Leu, Tyr, Phe and Pro. Positively charged amino acids (PCAA): His, Lys and Arg. Aromatic amino acids (AAA): Tyr and Phe. ND: not detected.

Table S5 [5]**.** Amino Acid profile of carp roe hydrolysates produced by pepsin, trypsin, and Alcalase

Amino acid (g/100 g protein)	Pepsin hydrolysate	Trypsin hydrolysate	Alcalase hydrolysate
**Essential amino acid**			
**Histidine**	2.74 ± 0.02	1.85 ± 0.02	2.60 ± 0.05
**Isoleucine**	6.05 ± 0.13	3.80 ± 0.19	5.74 ± 0.04
**Leucine**	9.57 ± 0.24	6.82 ± 0.09	9.05 ± 0.06
**Lysine**	6.81 ± 0.07	6.27 ± 0.06	6.83 ± 0.02
**Met + Cys**	2.7 ± 0.005	1.56 ± 0.04	2.7 ± 0.005
**Phe + Tyr**	7.95 ± 0.13	5.42 ± 0.05	7.56 ± 0.04
**Threonine**	4.92 ± 0.21	4.25 ± 0.02	4.98 ± 0.06
**Valine**	6.71 ± 0.01	4.78 ± 0.09	6.57 ± 0.01
**Nonessential amino acid**			
**Alanine**	7.74 ± 0.10	5.93 ± 0.03	7.49 ± 0.08
**Glycine**	3.46 ± 0.07	3.88 ± 0.04	3.91 ± 0.04
**Arginine**	6.23 ± 0.19	5.50 ± 0.09	5.77 ± 0.02
**Aspartic acid**	7.07 ± 0.36	6.56 ± 0.05	7.11 ± 0.09
**Glutamic acid**	14.1 ± 0.20	13.2 ± 0.25	15.3 ± 0.28
**Proline**	5.59 ± 0.08	5.39 ± 0.10	5.39 ± 0.06
**Serine**	6.04 ± 0.05	8.84 ± 0.07	7.49 ± 0.02
**TEAAs**	47.5 ± 0.02	34.7 ± 0.07	46.0 ± 0.15
**PDCAAS**	100	62.27	100

Cys, cysteine; Met, methionine; PDCAAS, Protein digestibility corrected amino acid score; Phe, phenylalanine; TEAA, total essential amino acids; Tyr, tyrosine

Table S6 [6]**.**The amino acid composition of the low molecular weight peptides from byproduct shrimp head of Solenocera crassicornis.

Amino Acid	Abbreviation	Ratio (g/100 g)
**Valine***	Val	4.10
**Leucine***	Leu	5.90
**Isoleucine***	Ile	3.94
**Lysine***	Lys	4.75
**Phenylalanine***	Phe	2.48
**Methionine***	Met	1.87
**Threonine***	Thr	3.57
**Histidine***	His	2.33
**Aspartic acid#**	Asp	7.65
**Serine#**	Ser	4.20
**Glutamic acid#**	Glu	9.56
**Glycine#**	Gly	6.38
**Alanine#**	Ala	3.79
**Tyrosine#**	Tyr	2.26
**Arginine#**	Arg	4.79
**Proline#**	Pro	2.30
**Essential amino acids***		28.94
**Non-essential amino acids#**		40.93
**Total amino acids**		69.87

Table S7 [7]**.** Amino acid composition of MCP from salmon skin.

Amino acid	No. residues/100 residues
**Glycine**	23.77
**Alanine**	6.59
**Serine**	4.23
**Proline**	9.79
**Valine**	2.94
**Threonine**	2.53
**Leucine**	4.64
**Isoleucine**	2.57
**Aspartic acid**	7.29
**Lysine**	5.66
**Arginine**	6.08
**Glutamic acid**	12.22
**Methionine**	0.03
**Histidine**	1.61
**Phenylalanine**	2.51
**Tyrosine**	0.03
**Hydroxy proline**	7.51

Table S8 [8]**.** Amino acid composition of the MCPs from the skin of chum salmon.

Amino Acid	No. Residues per 100 Residues
**Glycine**	23.77
**Glutamic acid**	12.22
**Proline**	9.79
**Hydroxyproline**	7.51
**Aspartic acid**	7.29
**Alanine**	6.59
**Arginine**	6.08
**Lysine**	5.66
**Leucine**	4.64
**Serine**	4.23
**Valine**	2.94
**Isoleucine**	2.57
**Threonine**	2.53
**Phenylalanine**	2.51
**Histidine**	1.61
**Methionine**	0.03
**Tyrosine**	0.03

Table S9 [9]**.** Amino acid composition of the collagen peptides derived from *Asterina pectinifera*.

Amino acid	(residues/1000 total amino acid residues)
**Glycine**	285.9
**Proline**	102.2
**Alanine**	91.7
**Hydroxyproline**	142.0
**Glutamic acid**	54.1
**Arginine**	23.2
**Aspartic acid**	29.3
**Serine**	40.1
**Lysine**	28.2
**Valine**	21.2
**Leucine**	17.4
**Threonine**	99.0
**Phenylalanine**	13.1
**Isoleucine**	7.6
**Hydroxylysine**	–
**Methionine**	23.6
**Histidine**	4.8
**Tyrosine**	16.6
**Cysteine**	–
**Total**	1000.0
**Hydrophilic amino acids (%)**	**30%**

Table S10 [10]**.** The amino acid composition of LP.

Amino acid	Concentration[a] (mg amino acid residues/g loach peptide powder)
**Aspartic acid**	69.7
**Glutamic acid**	133
**Serine**	28.1
**Glycine**	46.9
**Histidine**	31.1
**Arginine**	35.4
**Threonine**	37.7
**Alanine**	55.9
**Proline**	43.7
**Tyrosine**	27.9
**Valine**	29.4
**Methionine**	19.6
**Cystine**	0.2
**Isoleucine**	31.0
**Leucine**	55.9
**Tryptophan**	65.7
**Phenylalanine**	33.0
**Lysine**	53.7
**Total**	798

a: One gram of loach protein from 0 to 5 kDa fraction contained 850 mg of total estimated protein. Thus, the approximate yield of the identified amino acid residues (amino acids minus H_2O) accounted for 798/850, or approximately 94% of the nominal protein.

Table S11 [11]**.** Amino acids composition (%) of UGP and GPHs.

Amino acids	UGP	GPH-TF	GPH-A
**Asx**	4.41	3.74	3.95
**Ser**	7.02	6.54	6.80
**Glx**	9.20	7.94	7.8
**Gly**	15.74	15.19	15.12
**His**[1]	3.62	3.93	4.05
**Arg**	8.76	8.68	6.88
**Thr**[1]	13.40	12.55	12.96
**Ala**[2]	6.20	5.86	6.08
**Pro**[2]	4.25	5.28	5.20
**Cys**[2]	3.20	1.61	2.54
**Tyr**[1,2]	2.21	3.63	3.55
**Val**[2,]	3.50	4.71	4.31
**Met**[1,2]	1.99	2.24	2.84
**Lys**[1]	4.25	3.99	4.00
**Ile**[1,2,]	2.36	3.31	2.96
**Leu**[1,2]	6.84	6.69	6.42
**Phe**[1,2]	3.00	4.01	4.52
[1]**EAA**	37.67	42.28	41.84
[2] **HAA**	28.69	41.33	38.42

[1] = EAA, essential amino acids; [2] = HAA, hydrophobic amino acids; Asx, aspartic acid + asparagine; Glx, glutamic acid + glutamine; GPH-TF and GPH-A are GPHs produced using triggerfish and *B. mojavensis* strain A21 proteases, respectively.

References:

1. Liang, J.; Pei, X.-R.; Wang, N.; Zhang, Z.-F.; Wang, J.-B.; Li, Y. Marine Collagen Peptides Prepared from Chum Salmon (Oncorhynchus keta) Skin Extend the Life Span and Inhibit Spontaneous Tumor Incidence in Sprague-Dawley Rats. *Journal of Medicinal Food* **2010**, *13*, 757-770, doi:10.1089/jmf.2009.1279.
2. Ding, J.-F.; Li, Y.-Y.; Xu, J.-J.; Su, X.-R.; Gao, X.; Yue, F.-P. Study on effect of jellyfish collagen hydrolysate on anti-fatigue and anti-oxidation. *Food Hydrocolloids* **2011**, *25*, 1350-1353, doi:https://doi.org/10.1016/j.foodhyd.2010.12.013.
3. Harnedy, P.A.; FitzGerald, R.J. Bioactive peptides from marine processing waste and shellfish: A review. *Journal of Functional Foods* **2012**, *4*, 6-24, doi:https://doi.org/10.1016/j.jff.2011.09.001.
4. Dinel, A.-L.; Lucas, C.; Le Faouder, J.; Bouvret, E.; Pallet, V.; Layé, S.; Joffre, C. Supplementation with low molecular weight peptides from fish protein hydrolysate reduces acute mild stress-induced corticosterone secretion and modulates stress responsive gene expression in mice. *Journal of Functional Foods* **2021**, *76*, 104292, doi:https://doi.org/10.1016/j.jff.2020.104292.
5. Plowden, J.; Renshaw-Hoelscher, M.; Engleman, C.; Katz, J.; Sambhara, S. Innate immunity in aging: impact on macrophage function. *Aging Cell* **2004**, *3*, 161-167, doi:https://doi.org/10.1111/j.1474-9728.2004.00102.x.
6. Kim, M.-J.; Kim, K.-B.-W.-R.; Sung, N.-Y.; Byun, E.-H.; Nam, H.-S.; Ahn, D.-H. Immune-enhancement effects of tuna cooking drip and its enzymatic hydrolysate in Balb/c mice. *Food Science and Biotechnology* **2018**, *27*, 131-137, doi:10.1007/s10068-017-0278-9.
7. Pei, X.; Yang, R.; Zhang, Z.; Gao, L.; Wang, J.; Xu, Y.; Zhao, M.; Han, X.; Liu, Z.; Li, Y. Marine collagen peptide isolated from Chum Salmon (Oncorhynchus keta) skin facilitates learning and memory in aged C57BL/6J mice. *Food Chemistry* **2010**, *118*, 333-340, doi:https://doi.org/10.1016/j.foodchem.2009.04.120.
8. Xu, L.; Dong, W.; Zhao, J.; Xu, Y. Effect of Marine Collagen Peptides on Physiological and Neurobehavioral Development of Male Rats with Perinatal Asphyxia. *Marine Drugs* **2015**, *13*, 3653-3671, doi:10.3390/md13063653.
9. Han, S.-B.; Won, B.; Yang, S.-c.; Kim, D.-H. Asterias pectinifera derived collagen peptide-encapsulating elastic nanoliposomes for the cosmetic application. *Journal of Industrial and Engineering Chemistry* **2021**, *98*, 289-297, doi:https://doi.org/10.1016/j.jiec.2021.03.039.
10. You, L.; Zhao, M.; Regenstein, J.M.; Ren, J. In vitro antioxidant activity and in vivo anti-fatigue effect of loach (Misgurnus anguillicaudatus) peptides prepared by papain digestion. *Food Chemistry* **2011**, *124*, 188-194, doi:https://doi.org/10.1016/j.foodchem.2010.06.007.
11. Nasri, R.; Abdelhedi, O.; Jemil, I.; Daoued, I.; Hamden, K.; Kallel, C.; Elfeki, A.; Lamri-Senhadji, M.; Boualga, A.; Nasri, M.; et al. Ameliorating effects of goby fish protein hydrolysates on high-fat-high-fructose diet-induced hyperglycemia, oxidative stress and deterioration of kidney function in rats. *Chemico-Biological Interactions* **2015**, *242*, 71-80, doi:https://doi.org/10.1016/j.cbi.2015.08.003.
